# Therapeutic Potential of Astrocyte Transplantation

**DOI:** 10.1177/09636897221105499

**Published:** 2022-06-30

**Authors:** Nataly Hastings, Wei-Li Kuan, Andrew Osborne, Mark R. N. Kotter

**Affiliations:** 1 Department of Clinical Neurosciences, University of Cambridge, Cambridge, UK; 2 Wellcome-MRC Cambridge Stem Cell Institute, University of Cambridge, Cambridge, UK

**Keywords:** astrocyte, transplantation, neurodegeneration, injury, brain, spinal cord

## Abstract

Cell transplantation is an attractive treatment strategy for a variety of brain disorders, as it promises to replenish lost functions and rejuvenate the brain. In particular, transplantation of astrocytes has come into light recently as a therapy for amyotrophic lateral sclerosis (ALS); moreover, grafting of astrocytes also showed positive results in models of other conditions ranging from neurodegenerative diseases of older age to traumatic injury and stroke. Despite clear differences in etiology, disorders such as ALS, Parkinson’s, Alzheimer’s, and Huntington’s diseases, as well as traumatic injury and stroke, converge on a number of underlying astrocytic abnormalities, which include inflammatory changes, mitochondrial damage, calcium signaling disturbance, hemichannel opening, and loss of glutamate transporters. In this review, we examine these convergent pathways leading to astrocyte dysfunction, and explore the existing evidence for a therapeutic potential of transplantation of healthy astrocytes in various models. Existing literature presents a wide variety of methods to generate astrocytes, or relevant precursor cells, for subsequent transplantation, while described outcomes of this type of treatment also differ between studies. We take technical differences between methodologies into account to understand the variability of therapeutic benefits, or lack thereof, at a deeper level. We conclude by discussing some key requirements of an astrocyte graft that would be most suitable for clinical applications.

## Main Points

Astrocytic pathologies are found in many disorders, thus transplantation of healthy astrocytes can have therapeutic benefits. Regional and functional astrocyte heterogeneity has to be considered when choosing the optimal transplant source.

## Introduction

Cell therapies constitute an emerging class of therapeutic approaches to promote regeneration of damaged tissues. In particular, the brain presents a lucrative target since many neurological disorders result from the death of specific cell types; replenishing the cells of the central nervous system (CNS) aims to restore lost abilities and modify the course of a disease.

For example, neurons represent a valuable therapeutic tool to replenish the loss of specific neuronal subtypes in neurological disorders, and some benefits can be seen upon neuronal engraftment in Parkinson’s disease (PD) patients and models^1–5^. However, neuronal axons can reach up to 1 m in length in the healthy CNS, and even if the cell bodies survive and integrate well into the local host network with their shorter dendrites, there is little evidence supporting the ability of transplanted neurons (or neuron-differentiated stem cells) to send long axonal projections to their correct targets. In line with this, several groups observed that the functional benefit derived from the neuronal transplantation therapy cannot be explained by restoration of correct axonal circuits, but rather may be due to the secretion of protective factors and integration in local cellular networks at the site of implantation where they encourage plasticity within existing cells^6–10^.

Astrocytic processes, on the other hand, are well-positioned to physiologically integrate into the local cellular networks to take place of lost or diseased host astrocytes. Their ability to interact with and regulate multiple aspects of functionality of other cell types, such as neurons, makes it plausible that astrocytic replacement would have an effect on the astrocytic networks and beyond.

Stem cells have also been widely used for grafting. While some positive indications were obtained in several early studies^11,12^, results remain variable, and safety concerns persist as the precise mechanisms of action of stem cell grafts are uncertain^13^. The exact differentiation paths of these grafts within the host tissue are unpredictable, and stem cells are more likely to give rise to unwanted cellular phenotypes under the hostile conditions of the diseased CNS than pre-differentiated cells. This may at least partially account for the inconsistency of functional outcomes. Moreover, transplanted stem cells can remain undifferentiated and migratory^14^, which does not preclude the possibility of tumor development longer term. Tumorigenicity after stem cell transplantation has been described in several models^15,16^, and the short life span of common model organisms does not allow for predictions of how such grafts may behave in, for instance, stroke or PD patients, who are able to survive for decades after their diagnosis.

A growing body of literature highlights the importance of astrocytes in the healthy brain. These cells represent a cell population that forms complex networks capable of cross-talking to neuronal nets^17–19^. Astrocytic networks appear to act as nexus points interacting with, and regulating key aspects of functions of neurons^20,21^, oligodendrocytes^22,23^, microglia^24^, and the neurovascular unit^18,25^; and the presence of human-specific astrocyte types^26,27^ points toward potentially novel roles of these cells to be considered in the case of patient-specific grafts. Astrocyte-lineage cells also act as stem cells with neurogenic potential that can replenish neuronal populations, especially under inflammatory conditions^28,29^.

Moreover, astrocytic abnormalities have been described in association with, and sometimes as a cause of, various developmental and degenerative disorders ranging from autism^30^ and Down syndrome^31^ to multiple sclerosis^32,33^ and psychiatric conditions^34,35^. These findings call for an assessment of the utility of astrocyte transplantation as a clinical strategy. Experimental depletion of these cells, either pharmacologic or genetic, has shown that the lack of functional astrocytes can lead to depressive symptoms^36,37^, cognitive^38^ and motor^39,40^ impairment, seizures^41^, abnormal behaviours^41,42^, memory disturbance^43,44^, and neuronal death^40,45^ in the healthy animals, indicating that the healthy astrocytes are indispensable for the normal brain function. In the context of disease and inflammation, however, both positive and negative outcomes of astrocyte depletion have been reported^45–47^, suggesting that the roles of these cells are complex, and it is necessary to understand the protective pathways in greater depth.

Traditionally, neuronal and neural stem cell (NSC) replacement have been considered as the main avenue for the treatment of brain disorders since neuronal death is a prominent hallmark of many conditions of the brain, and a good number of detailed reviews on this topic exist^48–51^. At the same time, despite growing academic and commercial (e.g. AstranauTX, Astrocyte Pharmaceuticals Inc, and Kadimastem) interest in astrocyte-directed therapeutics, the literature on astrocyte transplantation is more limited compared to the neuronal counterpart, and opinions on its effectiveness diverge.

In the current review, we outline the disorders in which transplantation of astrocytes has been attempted so far. We describe examples of transplantation strategies employed in the following disorders, or the models of: amyotrophic lateral sclerosis (ALS), Parkinson’s, Alzheimer’s, and Huntington’s diseases, traumatic injury, and ischaemic stroke. We examine some common themes emerging across these pathologies—including immune milieu alternations, calcium signaling abnormalities, and mitochondrial dysfunction, and consider the mechanisms through which healthy astrocytes can therefore ameliorate these disorders.

A number of recent reviews discussed astrocyte-lineage cells in the context of transplantation as therapeutic and research modeling options. Transplantation of astrocytes and their progenitors has been reviewed in the context of ALS, and the importance of the location of the graft has been emphasised^52,53^. In PD, the dual beneficial versus harmful nature of astrocytic involvement has been considered, and some potentially protective genetic modifications of transplanted cells have been put forward^54^. Replacement of damaged cells, including astrocyte-lineage cells, after spinal cord injury (SCI), has been discussed, alongside the use of biomaterials to aid in cell guidance and integration into the host tissues^55,56^. Several reviews focused on the regenerative potential of stem cells and progenitors such as glial-restricted precursor cells (GRPs) capable of differentiating into astrocytes among other cell types as sources of cellular grafts^57–64^, or the ability of transplanted stem cells to interact with the host astrocytes and stimulate protective pathways in the latter^65–68^ in several conditions, including stroke, SCI, and Alzheimer’s disease (AD). In particular, protection of the blood-brain barrier (BBB) and the neurovascular unit by transplanted astrocytes has received attention in studies of neurodegenerative diseases^63^. Heterogeneity of astrocytic phenotypes (“neurotoxic” A1 and “neuroprotective” A2) and its influence on their ability to exert protective functions in disorders such as stroke have been considered^69^. Moreover, human chimeric mice, in which cell transplantation has been employed to model neurological diseases, allowed researchers to study disease mechanisms in highly translationally relevant models^70^.

The therapeutic benefits of astrocyte transplantation have been variable, and the reasons for these discrepancies have not yet been examined in depth, nor summarized in a single publication. We hypothesize that the discrepancies could be, at least in part, attributed to the astrocyte heterogeneity due to the technical differences in the astrocyte generation as well as the brain region (of cell origin and of the transplant location within the recipient). We take a closer look at the proposed roles of astrocytes in brain disorders and how these cells specifically become affected by various pathologies. Then, we outline the methodologies used to generate astrocytes, or precursor cells, for subsequent engraftment, in order to identify the most promising strategies that could be scaled up for future clinical applications. Overview of the full mechanisms of the listed pathologies beyond astrocytes (including neuronal pathology and an in-depth overview of the neurogenic potential of astrocytes) is outside of the scope of the current review, and interested readers are provided with a brief summary of each disorder supplemented with some useful references.

### Amyotrophic Lateral Sclerosis

ALS is a disorder in which benefits of astrocytic grafting are most clearly established as demonstrated by the outcomes of the phase II clinical trial for ALS (10 patients split across two cohorts) released in December 2020. The treatment involved intrathecal injection of astrocytes into the spinal cord and was found to be well-tolerated; it also reduced the disease progression rate in a significant and clinically meaningful manner (Table 1).

**Table 1.** Astrocyte Transplantation Strategies for the Treatment of ALS.

Disease or disease model and age at transplantation	Type of astrocyte-lineage cells or precursors transplanted	Site of transplantation within the CNS	Endpoint/treatment duration	Outcome summary	References
ALS—human adults, 10 patients (5 patients per cohort)	Pre-differentiated astrocytes derived from human embryonic stem cells—AstroRx® (Produced by Kadimastem); 100 × 10^6^ cells per patient in Cohort A, 250 × 10^6^ cells per patient in Cohort B	Intrathecal administration—spinal cord under local anesthesia	6 months for Cohorts A and B. Repeated treatment schedule (every 2–3 months) planned for the upcoming Cohort C	Phase I&IIa clinical trials, assessment of safety and preliminary efficiency. No treatment-related serious adverse effects (SAEs), no dose-limiting toxicities. Statistically significant reduction in the muscle function decline assessed by the average ALS Functional Rating Scale-Revised (ALSFRS-R) change for 3–4 months post-treatment compared to the pre-treatment period	^a^
ALS—SOD1^G93A^ rats, 90 day-old	GRPs derived from healthy rodent (rat or mouse) embryonic spinal cords	Cervical spinal cord ventral horn levels C4,5, and 6 bilaterally	Until end-stage, up to approx. 3 months post-transplantation (end stage was defined by the inability of rats to right themselves within 30s when placed on the side)	GRPs differentiated preferentially into astrocytes; reduced microgliosis, slower decline in forelimb motor control and respiratory function, reduced motor neuron loss. GRPs deficient in GLT1 served as a negative control and did not show positive effects.	Lepore et al.^71^
ALS—SOD1^G93A^ mice, 50- to 60-day old	Human GRPs (Q-Cells®) derived from fetal forebrain	Cervical spinal cord ventral horn levels C4 and 5 bilaterally	Until end-stage, up to approx. 3 months post-transplantation (end stage was defined by the inability of mice to right themselves within 30s when placed on the side)	Human GRPs differentiated preferentially into astrocytes, up to 10% of cells continued to proliferate 3 months post-engraftment. No therapeutic benefit achieved.	Lepore et al.^72^
ALS—SOD1^G93A^ mice, 90 day-old	Human iPSCs line 201B7 clone selected for its low tumorigenicity, pre-differentiated into glial-rich neural progenitors (GRNPs) by BMP4+LIF treatment	Lumbar spinal cord ventral horn levels L3 and 4 bilaterally	Until end-stage, up to approx. 2.5 months post-transplantation	Modest lifespan prolongation, transient improvement of the lower limb function.	Kondo et al.^73^
ALS—SOD1^G93A^ mice, 67 ± 2 days-old, or 2 injections at 67 ± 2 and 97 ± 2 days of age	Human astrocytes derived from human ESCs (two stem cell lines: HADC100 and NCL-14) through astrocyte progenitor cell (APC) stage	Injection into the CSF through cisterna magna	Until end-stage, approx. 2 months post-first transplantation point	Trends toward longer survival, improved motor performance on the rotarod, significantly delayed disease onset; two injections are more efficient than one.	Izrael et al.^74^
ALS—SOD1^G93A^ rats, 2 injections at 50 ± 2 and 70 ± 2 days of age	Same as above	Intrathecal injection by lumbar puncture in the subarachnoid space between L5 and L6 (most relevant to the human administration method)	Until end-stage, up to 5.5 months post-first transplantation point	Trends toward longer survival, significantly delayed disease onset, treated rats maintained their body weight significantly longer, improved motor performance on the rotarod and in the grip strength test; no tumors formed.	Izrael et al.^74^

ALS: amyotrophic lateral sclerosis; BMP4: bone morphogenic protein 4; CNS: central nervous system; GLT1: glutamate transporter 1; GRPs: glial-restricted precursors; GRNPs: glial-rich neural progenitors; CSF: cerebrospinal fluid; ESCs: embryonic stem cells; LIF: leukemia inhibitory factor; SOD1: superoxide dismutase 1.

a https://clinicaltrials.gov/ct2/show/NCT03482050; https://www.kadimastem.com/post/kadimastem-announces-promising-results-of-cohort-a-of-its-phase-1-2a-clinical-trial-in-als; https://www.kadimastem.com/post/encouraging-results-of-cohort-b-of-its-phase-1-2a-clinical-trial-of-astrorx-for-als.

ALS usually affects adults with an average age of diagnosis of 55 years and an incidence of about two per 100 000 people. Only 5% to 10% of all ALS cases run in families, as opposed to sporadic onset^75,76^. Superoxide dismutase 1 (SOD1) gene mutations are most commonly associated with ALS accounting for 20% of familial cases^77^. This mitochondrial enzyme acts by scavenging superoxide radicals to protect cells from excessive amounts of reactive oxygen species (ROS). Other genes such as a DNA-binding protein TDP-43/TARDPB and C9ORF72 have also been implicated in ALS pathogenesis, suggesting that multiple aetiological pathways have the capacity to lead to the eventual ALS manifestation—profound loss of upper and lower motor neurons in the spinal cord and brain leading to eventual paralysis^75,78,79^.

Interestingly, even though the neuronal pathology is well-described, profound changes in astrocytes accompany or even precede the disease. Causal effects of astrocytic aberrations in the ALS pathology are demonstrated by the motor dysfunction and motor neuron degeneration initiated after transplantation of the SOD^G93A^ harboring GRPs^80^ or ALS patient-iPSC-derived astrocytes^81,82^ into the spinal cord of the wild-type mice. Following this line of evidence, healthy astrocyte transplantation showed a beneficial effect upon engraftment into the spinal cords of SOD1 mutant mice and rats^71,73,74^, prolonging survival time and diminishing the disease progression rate in these animals (Table 1). In accordance with these data, astrocyte-specific decrease in the mutant SOD1 load attenuated disease progression in mouse ALS models^83,84^.

In addition to the non-cell autonomous effects on motor neurons caused by SOD mutations in astrocytes, ALS-associated mutations induce drastic changes in astrocyte biology, at least *in vivo*, including reactive astrocytosis, also known as astrogliosis, as evidenced by the increased GFAP expression and process hypertrophy^85–90^, and ultimately astrocyte degeneration and apoptosis^91^. These can result from either, or a combination of, mutations within astrocytes themselves, or from altered functions of surrounding neurons and other cells such as microglia. Curiously, some of the proliferating GFAP-positive cells in ALS may not be true astrocytes, but aberrant glial cells with an astrocyte-like phenotype of microglial origin, which may not be sufficient to substitute for the loss of the true astrocytic functions while contributing to the pro-inflammatory milieu generation^92,93^.

As suggested by the fact that SOD is an enzyme involved in mitochondrial protection from excessive levels of ROS, oxidative stress alongside mitochondrial damage are well-established hallmarks of ALS^94–96^, which is also true in cases of ALS caused by other mutations^97–99^. Since astrocytes are a major source of the antioxidant glutathione (GSH) in the brain^100,101^, oxidative damage to astrocytes is likely to make the brain milieu more vulnerable to ROS. Mitochondrial abnormalities, elevated levels of inducible nitric oxide synthase (iNOS), and ultimately increased levels of ROS in astrocytes are associated with motor neuron degeneration^102–104^. Since mitochondrial functions are closely associated with calcium signalling^105–107^, it is not surprising that altered calcium homeostasis has been observed in astrocytes carrying ALS-linked mutations^108–110^. Abnormally elevated intracellular calcium levels in response to stimuli such as ATP^109^ could result in caspase activation with subsequent astrocytosis^111^. Enhanced SNARE-dependent exocytosis of the vesicles containing microRNA, glutamate, and ATP may also contribute to the neuronal toxicity of the mutant astrocyte-conditioned medium^109,112,113^.

Several secreted factors can also be responsible for such neurotoxic effects, including upregulated IFNγ, IL6, prostaglandin D2, tumor necrosis factor α (TNFα), and TGFβ that are released in the extracellular milieu^114–116^. Moreover, elevated extracellular glutamate levels were also found in ALS models where oxidative stress and potentially other changes in SOD1 mutant astrocytes result in downregulation of glutamate transporters^117,118^, initiating excitotoxic cascades in the neighboring motor neurons. Activation of caspase-3, a marker of apoptosis, in astrocytes can also downregulate glutamate transporter EEAT2 expression^119^, thereby contributing to the increased extracellular glutamate accumulation and excitotoxicity to perpetuate the vicious cycle. On the other hand, healthy astrocytes harboring a “younger” phenotype, such as the hESC-derived cells used for grafting in a murine model of ALS, exhibit a more protective secretory profile by supplying GDNF, VEGF, osteopontin, and CXCl-16 chemokine, which stimulate neuronal survival and regeneration, as well as matrix metalloproteinase inhibitors TIMP-1 and 2, in order to preserve supportive extracellular matrix (ECM) composition and suppress immune cell infiltration^74^.

Considering that the ALS-associated mutations affect all cells in the brain, selective vulnerability of motor neurons, but not other neuron types, co-cultured with SOD1 mutant astrocytes is intriguing. Activation of LT-βR by theTNF superfamily member LIGHT triggers a motor neuron-selective death pathway^120^, and so does the activation of FasL^121,122^. Both of these pathways are potentiated by diseased astrocytes through secretion of IFNγ^120^ or ATP^123^, respectively. Additionally, NGF, secreted by astrocytes under inflammatory conditions such as those found in ALS^104,124^, promotes cell survival in cells expressing both TrkA and p75^NTR^,^125^ but if TrkA is absent, cell death results^126,127^. Although motor neurons are normally devoid of both, in ALS re-expression of p75^NTR^ becomes evident^128–130^, which may contribute to this selective degeneration.

In this context, replacement of healthy astrocytes is a promising approach for the treatment of ALS. Wild-type astrocytes can help reduce excitotoxicity by removing excessive glutamate, provide lactate for surrounding neurons, reduce oxidative stress, and secrete anti-inflammatory factors that are beneficial for both diseased neurons and astrocytes in the host tissue. Effective healthy astrocyte replacement should be able to improve the muscle function and ultimately prolong the patient’s lifespan, with Kadimastem’s AstroRx offering a potential candidate for further scaling up of this approach.

### Parkinson’s Disease

PD is a progressive neurodegenerative condition that shares some pathophysiological similarities with ALS, including abnormal protein aggregation and mitochondrial dysfunction^131–133^. Moreover, up to a third of all ALS patients experience symptoms of Parkinsonism^134^, and the co-occurrence of the two pathologies is especially obvious in Lytico-bodig disease and in the ALS-parkinsonism-dementia complex^135^. PD is the second most common neurodegenerative condition that typically affects older adults, with 96% of cases diagnosed after the age of 50, with an incidence of almost 2,000 per 100 000 people over the age of 80^136^,^137^.

PD is a clinically heterogenous disorder characterized by the loss of dopaminergic neurons^138^ accompanied by reactive changes in astrocytes and microglia^139^ in the nigrostriatal system. Loss of the nigrostriatal dopaminergic innervation results in persistent tremor, bradykinesia, rigidity, and postural instability^140,141^. However, it becomes apparent that many other systems are affected by PD including autonomic and cognitive dysfunctions^142^. It has been suggested that the earliest signs of PD may start within the gastrointestinal system where resident neurons^143,144^ and astrocyte-lineage-related cells (enteric glial cells [EGCs])^145^ become affected. Accumulation of α-synuclein protein aggregates known as Lewy bodies is a common neuropathological finding that tends to spread in a stereotypic pattern known as Braak stages—hindbrain structures such as the brain stem and midbrain develop a higher protein aggregate load earlier in disease while forebrain structures remain relatively unaffected until later disease stages^146–148^. Current treatments include dopamine replacement and deep brain stimulation (DBS), which only provide symptomatic relief, while serious side effects including hallucinations and drug-induced dyskinesias often limit the therapeutic benefits of these approaches^138^. Interestingly, the mechanism of action of DBS may include stimulation of chemical transmitter release from astrocytes^149^.

Human astrocyte transplantation has been trialed in rodent models of PD with promising results (Table 2).

**Table 2.** Astrocyte Transplantation Strategies for the Treatment of PD.

Disease or disease model and age at transplantation	Type of astrocyte-lineage cells or precursors transplanted	Site of transplantation within the CNS	Endpoint/treatment duration	Outcome summary	References
PD—6-OHDA injected (left striatum) Sprague-Dawley rats, adult	Human mesenchymal stem cells (MSCs) pre-differentiated into astrocyte-like cells	Left striatum, 6 weeks post-lesion	120 days (4 months) after grafting)	Reduced rotational behavior, enhanced density of dopaminergic fibers.	Bahat-Stroomza et al.^150^
PD—6-OHDA injected (right striatum) Fisher 344 male rats, adult	GDAs, human or Fisher 344 hPAP transgenic rat, treated with either BMP4 or CNTF	Right striatum, 4 weeks post-lesion	7 weeks post-lesion (3 weeks post-transplantation); 2 weeks post-treatment for behavior analysis	GDA^BMP4^ (but not GDA^CNTF^) transplantation benefits: improvement in left paw usage, reduced rotational behavior, increased TH labeling (dopaminergic neuron marker) in the right striatum and protection of parvalbumin-expressing GABAergic interneurons, increased synaptophysin expression.	Proschel et al.^151^
PD—6-OHDA injected (right side of substantia nigra and the median forebrain bundle), Sprague-Dawley female rats, adult	NPCs, from mouse embryonic ventral midbrain) alone or in combination with cortical or ventral midbrain rodent postnatal day 5- to 7-exctracted and cultured astrocytes; or with ventral midbrain astrocytes transduced with Nurr1+Foxa2 transcription factors	Striatum bilaterally	6 months post-transplantation	Co-grafting NPCs with ventral midbrain astrocytes showed the greatest amount of benefits in terms of reduction of rotational behavior to levels comparable with controls and number of NPCs differentiating into dopaminergic neurons.	Song et al.^152^

PD: Parkinson’s disease; BMP4: bone morphogenic protein 4; CNS: central nervous system; CNTF: ciliary neurotrophic factor; GDAs: glial precursor derived astrocytes; hPAP: human placental alkaline phosphatase; NPCs: nNeural precursor cells; 6-OHDA: 6-hydroxydopamine; TH: tyrosine hydroxylase.

A unique population of astrocytes matured from GRPs through the exposure to bone morphogenic protein 4 (GRPs^BMP4^) rescued motor symptoms, which was accompanied by an increase in striatal dopamine production and neuronal survival^151^. Other methods of human astrocyte lineage cell generation, such as differentiation from the adult bone marrow mesenchymal stromal cells, yielded similar behavioral improvements^150^. In another study, modified astrocyte-lineage cells destined for the striatal graft were engineered to deliver tyrosine hydroxylase (TH), which is normally expressed by neurons, enhanced local dopamine synthesis, and improved motor coordination in a rat model^153^. Finally, co-grafting of astrocytes alongside neural progenitor cells into the striatum also led to an enhanced behavioral recovery in a mouse model of PD that surpassed the therapeutic benefit of grafting these progenitors alone^152^.

Perhaps the most compelling evidence of astrocyte involvement in PD was shown by the selective astrocytic expression of mutant A53T α-synuclein, associated with a familial form of the disorder, which led to profound dopaminergic and motor neuron degeneration accompanied by microglial activation within the hindbrain^154^. Moreover, co-culture of healthy neurons with patient-derived astrocytes differentiated from iPSCs led to neurodegeneration and α-synuclein accumulation^155^, showing that PD-linked mutation in astrocytes alone is sufficient for profound pathology in astrocytes and neurons to develop. It is therefore evident that these cells play an important role in PD progression. It is noteworthy that, under healthy conditions, mesencephalic regions such as SNpc (which are more vulnerable to PD-inducing insults) may be less dense in astrocytes compared to neighboring regions^156^, pointing at the neuroprotective effects of astrocyte presence. Curiously, two drugs currently being explored in human PD clinical trials, zonisamide^157^ and rotigotine^158^, have been shown to increase astrocytic proliferation and stimulate secretion of neuroprotective factors from these cells.

Despite α-synuclein being a predominantly neuronal protein, α-synuclein aggregates in other cell types including astrocytes have been reported^148^, and specific upregulation of α-synuclein was seen in astrocytes derived from patient iPSCs carrying LRRK2 mutation (kinase involved in autophagy and associated with an autosomal dominant form of PD)^155^. Astrocytes carrying LRRK2 mutations that were derived from patient iPSC lines also showed decreased astrocytic marker expression and complexity, and these cells produced higher levels of ROS^159^. In addition to the cell-autonomous changes in PD astrocytes, these cells are capable of taking up the misfolded α-synuclein from neurons through endocytosis^160–163^, which can result in pathological activation of the former^164^. α-synuclein aggregates taken up by astrocytes can be cleared via the lysosomal pathway^165,166^, but excessive protein build-up resulting from the increased α-synuclein release^167^, or insufficient clearance^165^, can lead to mitochondrial stress^168^, autophagy dysfunction^155,166,169^, ER-Golgi system stress^170^, and eventual astrocyte apoptosis^169–172^. Importance of the mitochondrial disbalance in PD pathogenesis is further emphasized by the fact that PINK1, an autophagy-related gene that is associated with familial PD forms and is predominantly active within astrocytes as opposed to neurons^173^, encodes a mitochondrial kinase. Mutations in this protein can lead to defective astrocytic proliferation and ATP levels, heightened ROS levels, and a decreased ability to uptake glucose as well as to lower growth factor receptor expression^174^. DJ-1 is another mitochondrial-stabilizing gene whose deletion causes a familial PD form, and its deletion negatively affected astrocytic mitochondrial function and the ability of these cells to protect astrocyte-neuron co-cultures against toxic insults^175^.

In response to cellular stress caused by α-synuclein^164^ and parkinsonism-inducing neurotoxins^176–180^, astrocytes undergo reactive changes^181^ resulting in neuroinflammation. Reactive astrocytosis does not only prevent these cells from secreting trophic factors like GDNF family of ligands, BDNF, NT3, and mesencephalic astrocyte-derived neurotrophic factor (MANF)^152,182–185^, but also stimulates release of pro-inflammatory cytokines such as IL1β^186^. Consistently, it has been noted that differentiated astrocytes used for transplantation express trophic factors that are known to be neuroprotective, which can at least partially account for the benefits of the transplantation^151,152^.

Transplanted astrocytes, therefore, can provide multiple benefits in PD by replenishing the resident astrocytic pool in place of the apoptotic cells, secreting neuroprotective factors, restoring potassium buffering, degrading α-synuclein through lysosomal pathways, and reducing oxidative stress by supplying ROS-scavenging enzymes. Artificial expression of transcription factors (Nurr1 and Foxa2) in astrocytes co-grafted with stem cells promoted a non-reactive phenotype of the astroglia^152^, suggesting that bioengineering approaches can ensure that the cells maintain the beneficial phenotype even in the presence of ROS and pro-inflammatory cytokines among other PD-associated stressors. Effective healthy astrocyte replacement in PD could be able to address motor and/or non-motor symptoms of this disorder. While most studies focused on the motor manifestations such as tremor and rigidity, up to 50% of people with PD report the non-motor symptoms, including memory issues, sleep disturbance, and depression, to be the major determinants of their quality of life^187^. Recalling that the experimental loss of astrocyte function in the cortical areas and the hippocampus result in memory disturbance^43,44^ and depressive symptoms^36,37^, inclusion of the non-motor symptoms of PD in the efficacy assessment of pre-clinical and clinical PD trials of astrocyte-centered therapies could prove fruitful and open new avenues complimentary to dopamine replacement approaches.

### Alzheimer’s Disease

AD is the most prevalent neurodegenerative disorder that shares important similarities with the pathogenesis of PD. These similarities include a progressive, stereotypic pattern of misfolded protein accumulation. It starts within the entorhinal cortex and medial temporal structures and gradually spreads to the basal ganglia^188,189^. Amyloid-β plaques derived from the amyloid precursor protein (APP), and hyperphosphorylated tau-containing neurofibrillary tangles are hallmarks of AD found extra- and intracellularly, respectively^190^. A number of tauopathies including frontotemporal dementia (FTD) are also associated with the tau tangles and lead to certain symptoms similar to those of AD^191,192^. Interestingly, amyloid-β and α-synuclein pathologies co-exist in up to 50% of AD patients^193–196^, suggesting that common cellular abnormalities, including those found in astrocytes, are likely to be found both in AD and PD.

Similarly to PD, AD typically manifests later in life, affecting around 10% of over 65-year-olds with disease prevalence strongly correlating with age^197–199^. Memory loss is an early symptom of AD^200^, but as the disease progresses, other neurological functions become affected and symptoms such as speech impairment and lack of motor coordination become prominent. Heritability of this disorder is estimated to range from 60% to 80%, suggesting an important role for genetic factors^200,201^. Mutations in the APP gene can predispose the amyloid-β protein to misfold and are often found in association with the familial form of the disorder. PSEN1 and PSEN2 are also commonly associated with AD and encode for the proteins in the γ-secretase complex, which is necessary to cleave amyloid-β from its precursor APP^200^. The ε4 allele of apolipoprotein (APOE4), a major cholesterol carrier, represents the strongest risk factor for sporadic late-onset AD^202^, and it is noteworthy that astrocytes show the highest degree of apolipoprotein expression among all CNS cell types^203^. Current AD treatment strategies are purely symptomatic; these include cholinesterase inhibitors, which aim to increase acetylcholine levels, and NMDA receptor antagonist memantine, which counteracts excitotoxicity^204^.

An interesting approach to transplantation has been attempted in a rat model of AD generated by the infusion of a toxic form of amyloid-β (1-42 peptide), where autologous EGCs that are related to astrocytes were harvested for grafting from animals’ own appendices. EGCs were delivered into cerebral ventricles, from which they were observed to migrate toward the amyloid plaques within the brains. These cells were not only able to reduce the plaque load, but also the cytokine profile in the treated brains was shifted toward a more anti-inflammatory phenotype with a significant decrease in TNFα, PGE2, and IL6, and increase in NGF, BDNF, and GDNF. Moreover, memory and learning skills were improved by the treatment^205^. These data are corroborated by the study conducted in a mouse transgenic model of FTD harboring human P301S tau, in which transplantation of NPC-differentiated astrocytes into the cortical gray matter reversed cortical neuron loss^206^ (Table 3).

**Table 3.** Astrocyte Transplantation Strategies for the Treatment of AD.

Disease or disease model and age at transplantation	Type of astrocyte-lineage cells or precursors transplanted	Site of transplantation within the CNS	Endpoint/treatment duration	Outcome summary	References
AD—Sprague-Dawley male rats infused with amyloid-β42 through a mini-osmotic pump device into the right cerebral ventricle for 20 days, adult	EGCs isolated from appendices of the same rats (appendices isolated during cannula insertion into the cerebral ventricle) and cultured for 20 days	Right cerebral ventricle through the same cannula left after the amyloid-β42 infusion	8 weeks	Autologous EGCs increased neurotrophin release and neurogenesis, reduced inflammatory cytokine secretion, induced amyloid-β plaque degradation in the cortex and hippocampus, and significantly ameliorated cognitive deficits as assessed by Morris water maze and object recognition tasks.	Esposito et al.^205^
Tauopathy / FTD—P301S tau mice, 8 weeks-old	NPCs were isolated from the cortices of the eGFP-expressing neonatal mice; astrocytes were differentiated from NPCs by BMP4 treatment	Cortical gray matter, one hemisphere per animal	4 or 12 weeks	Both NPCs (which preferentially differentiated into astrocytes) and pre-differentiated astrocytes significantly reduced neuronal loss.	Hampton et al.^206^

AD: Alzheimer’s disease; BMP4: bone morphogenic protein 4; CNS: central nervous system; EGCs: enteric glial cells; eGFP: enhanced green fluorescent protein; FTD: frontotemporal dementia; NPCs: neural precursor cells.

Multiple pathological changes in astrocytes have been observed in AD and animal models of this disease^207^. Reactive astrogliosis and disruption of the astrocytic domain organization occur in an AD mouse model even before the appearance of the amyloid plaques^208,209^; similar evidence of astrocyte activation and degeneration was found in the brains of AD patients^210^. Indeed, predominance of the pro-inflammatory (e.g. IL1, TNFα) versus anti-inflammatory cytokines are well-documented hallmarks of AD^211^. Alterations in the surface-expressed receptors can take place in AD, such as in the case of EphB2 receptor upregulation on hippocampal astrocytes that can downregulate synaptic plasticity^212^. While reactive astrocytes surrounding amyloid-β plaques may be protective at initial disease stages, astrocytic activation increases linearly with cognitive decline^213^, likely contributing to the disease progression. Accordingly, suppression of astrocyte activation through inhibition of the JAK-STAT3 cascade was found to improve outcomes in a mouse model of AD^214^.

Interestingly, even though astrocytes surrounding plaques were found activated and hypertrophic in triple-transgenic (3xTg-AD) mice (harboring mutations in APP, presenilin, and tau), astrocytes distant from the plaques, or those analyzed at stages prior to plaque formation, were found to have dystrophic branches with reduced complexity^208,209^. Moreover, atrophic astrocytes were also found in the hippocampi of PDAPP mice exhibiting high levels of human APP expression^215^. Regional heterogeneity of astrocytic response has been observed with entorhinal cortex exhibiting less astrocytic activation compared to other regions such as hippocampus^208^, which may underlie selective vulnerability of certain brain regions to the AD-related degeneration.

APP, unlike tau, is expressed not only by neurons but also by astrocytes^216^; at the same time, healthy astrocytes do not express β-secretase (BACE1), a key enzyme necessary to cleave amyloid-β from APP. However, inflammation, or chronic stress in the context of AD can induce astrocytic BACE1 expression in AD, thereby contributing to the amyloid-β load^216,217^. Release of amyloid-β can have cell-autonomous effects that compromise astrocytic viability^218^, and also activate microglia^219^. In addition, formation of amyloid-β oligomers is able to induce ROS release from astrocytes and trigger the loss of protective transcriptional activity of STAT3 in neurons^220^. Accordingly, increased oxidative stress was observed in astrocytes harboring tau mutations associated with FTD^221^. Considering the close connection between the ROS production and abnormal mitochondrial function, it is not surprising that multiple alterations in mitochondria-related genes were found in astrocytes from AD patients’ brains compared to the healthy elderly controls^222^.

In AD, astrocytes have been shown to be capable of clearing misfolded amyloid proteins through endocytic mechanisms and promote its elimination through the lysosomal pathway^223^ or transcytosis and cerebrospinal fluid (CSF) clearance^218^. Such process is hampered by mutations in the APOE4 allele in astrocytes, which is associated with the excessive endosomal acidification, defective autophagy, and ultimately lack of sufficient amyloid-β clearance through the endosome-lysosome pathway^224^.

Hence, astrocyte grafting can aid in clearance of senile plaques, reduce pro-inflammatory cytokine concentration, supply antioxidants and enhance neurotrophic factor release, as a neuroprotective mechanism in response to the amyloid-β42 challenge^225^. It can also substitute for the atrophic and apoptotic cells in the astrocytic syncytium which could buffer abnormally elevated calcium levels in the astrocytic network. Effective healthy astrocyte replacement in AD could improve the cognitive function or reduce the rate of the functional and cognitive decline.

### Huntington’s Disease

Huntington’s disease (HD) is caused by a single gene mutation—a polyglutamine (CAG repeat) expansion on the N-terminal region of huntingtin gene (*HTT*), in which the size of the expanded region inversely correlates with the age of disease onset^226^. Similar to other neurodegenerative proteinopathies discussed above, the pathological hallmark of HD is the aggregation of the mutant huntingtin protein (mtHtt), which can trigger the progressive neurodegeneration, particularly of the striatal GABAergic medium spiny neurons (MSNs), although other areas such as cortex also become affected^227^. No cure exists except palliative treatments that aim to alleviate the involuntary movements, or chorea, by reducing dopaminergic neurotransmission, and to suppress psychiatric manifestations^228,229^.

The huntingtin protein is naturally expressed in various cell types including cells outside of the brain^230–233^. Astrocytic pathology has been implicated in HD as shown by the study in which specific expression of mtHtt in this cell type in mice was sufficient to induce profound pathology, including motor abnormalities, body weight loss, and lower life expectancy^234^. *In vitro* astrocyte-neuron co-cultures demonstrated that astrocytes harboring mtHtt increased vulnerability of neurons, while wild-type astrocytes protected neighboring neurons from excitotoxicity^231^. Even more strikingly, while neurons differentiated from patient-derived iPSCs were phenotypically normal and survived in the adult mouse brain, astrocytes generated from the same cells showed cytoplasmic vacuolation under basal cell culture conditions, suggesting that *HTT* mutations can induce astrocytic aberrations in a cell-autonomous manner, and that astrocytes are highly sensitive to mtHtt accumulation^235^. Human glial progenitor cells (hGPCs) expressing mtHtt fail to differentiate into mature GFAP-expressing astrocytes in the rodent brain^236^. Accordingly, specific reduction of mtHtt load in astrocytes slows down disease progression in a mouse model^237^.

It is therefore not entirely surprising that transplantation of healthy astrocytes can alleviate HD disease phenotype in transgenic mice. Indeed, hGPCs comprised of astrocyte-biased precursors, when engrafted neonatally into the striatum of HD mice, successfully differentiated into astrocytes (or persisted as precursors) and delayed motor and cognitive deterioration. Importantly, striatal atrophy was also reduced by the astrocyte transplantation (Table 4). On the other hand, reverse engraftment of mtHtt-bearing precursors into healthy rodents resulted in the manifestations of HD with impaired motor coordination^238^.

**Table 4.** Astrocyte Transplantation Strategies for the Treatment of HD.

Disease or disease model and age at transplantation	Type of astrocyte-lineage cells or precursors transplanted	Site of transplantation within the CNS	Endpoint/treatment duration	Outcome summary	References
HD—R6/2 (heterozygous transgenic for the 5’-end of the human HTT gene) x rag1-/- (immunodeficient) mice, postnatal day 1 (P1)	Astrocyte-biased human fetal glial precursor cells (GPCs) were isolated from the forebrain tissue (18–22 week-gestational age)	Striatum bilaterally	8–18 weeks depending on the test	Integrated human cells did not express mHTT aggregates; treated mice survived significantly longer, showed less striatal volume loss, exhibited improved motor performance assessed by the rotarod and gait tests, and better cognitive performance analyzed by the T-maze test. Striatal neurons showed improved electrophysiological properties, and reduced striatal potassium levels were found in the presence of transplanted glia.	Benraiss et al.^238^

HD: Huntington’s disease; CNS: central nervous system; HTT: huntingtin; mHTT: mutant huntingtin.

One documented astrocytic pathology associated with the *HTT* mutation and symptom onset in HD models is the reduction in inwardly rectifying potassium channel Kir4.1 expression, which alters the electrophysiological profile of astrocytes and makes surrounding neurons more prone to excitotoxic death.

Concomitant with the Kir4.1 downregulation, astrocytic and neuronal depolarization alongside elevated extracellular potassium levels were observed in a mouse HD model. Artificial AAV-mediated expression of Kir4.1-GFP in astrocytes in this model attenuated the characteristic MSN hyperexcitability associated with HD, as well as improved motor function of the animals and increased their life span^239^. To further exacerbate excitotoxicity caused by Kir4.1 downregulation, deficit in glutamate transporters, especially GLT-1, was observed in mouse models and cultured astrocytes harboring mtHtt, which can further contribute to MSN hyperexcitability by increasing glutamate concentrations within striatum^231,240–244^. Nuclear mtHtt inclusions are able to directly suppress GLT1 expression through interaction with and inhibition of its promoter^234^.

Post-mortem analysis of HD brains revealed morphological changes and increased astrocytosis, which correlated with disease progression^243^. Inflammation and astrocyte activation are likely to play a role in HD pathogenesis as higher levels of NFκB were found in astrocytes from HD patients and mouse models, and systemic inflammatory stimuli (e.g. LPS) elicited more prominent cytokine release from such cells. Accordingly, inhibition of the IκB kinase-NFκB pathway improved neuronal survival and ameliorated motor and cognitive deficits in the R6/2 mice^245^. JAK-STAT signaling represents another canonical pathway involved in astrocytic activation and subsequent microglial recruitment in HD. Surprisingly, blockade of this pathway increased mtHtt aggregation without affecting neuronal survival, suggesting that some aspects of astrocytic reactivity serve as a protective compensatory mechanism aimed at elimination of excessive misfolded protein^246^. Reactive astrocytes are also able to exert neuroprotective effects by promoting mtHtt degradation through upregulation of autophagy, lysosome, and proteasome-related genes^247^, which may also be a cell-autonomous, protective mechanism underlying lower mtHtt accumulation in this cell type^232^. However, this may only represent an initial compensatory response that subsides over time, as progressive ubiquitin-proteasome system decrease was shown in neurons and astrocytes of aging mice^232,233^.

As expected, neurons are not the only cells whose excitability is altered in HD. A mouse HD model has demonstrated altered spontaneous calcium signaling in striatal astrocytes that had, on average, diminished frequency, amplitude, and duration compared to wild-type animals. At the same time, astrocytes responded more robustly to the activity of cortical neurons due to excessive glutamate accumulation resulting from an insufficient glutamate uptake and deficiency in potassium channels^240,248^. To corroborate these data, specific reduction in calcium signaling in wild-type mice by transducing striatal astrocytes with a construct encoding a calcium pump PMCA2, which extrudes calcium from the cytosol, leads to excessive self-grooming behavior reminiscent of that observed in R6/2 mice^249^. Furthermore, diminished calcium signaling was found in R6/2 HD mouse model, which preceded later-stage severe motor dysfunction associated with the striatal tissue loss^42^.

In addition to abnormal cytosolic calcium signaling, mitochondrial calcium dynamics are likely to contribute to HD pathology. It is noteworthy that mitochondria of striatal astrocytes (and neurons) are less capable of calcium buffering compared to cortical cells, providing another insight into the selective vulnerability of this brain region^250^. Moreover, striatal mitochondria present with reduced mitochondrial respiratory capacity compared to their cortical counterparts, and antioxidant (N-acetylcysteine) treatment ameliorated some motor symptoms in an HD mouse model^251^.

Multiple other astrocytic genes were found to be altered in HD models, which results in suppressed BDNF secretion, perturbed calcium signaling pathways, and astrocyte activation among other pathological changes^252,253^. Since astrocytosis is known to be associated with neurotoxicity, pro-inflammatory cytokine profile, and even eventual astrocyte apoptosis, as discussed above for other known disorders, these factors could be expected to contribute to MSN death and other manifestations of HD. Effective healthy astrocyte replacement in HD could improve the cognitive function or reduce the rate of cognitive decline, reduce psychiatric symptoms, and/or help control the motor manifestations of this disorder, thus increasing the functional independence of patients.

### Traumatic Injury to the CNS

SCI and traumatic brain injury (TBI) are among the leading causes of preventable disability in the younger population. Nearly half of the SCI incidents occur between ages 16 and 30, while only less than 1% of the affected patients make a full recovery^254,255^. Globally, TBI is about twice as prevalent as SCI with the numbers of cases of approximately 760 per 100 000 and 370 per 100 000, respectively^256^. Even though vehicle collisions, sport-related traumas, and physical assault are common causes of CNS injuries in younger patients, falls account for more injuries in the older population^255^, sometimes becoming a co-morbidity of another degenerative disorder affecting motor functions such as those described above.

Unlike neurodegenerative conditions where cellular dysfunctions are found in multiple brain areas, traumatic injury in cases such as spinal fracture-dislocation represents a localized area of damage and inflammation. This makes cell transplantation therapies more applicable, although many cases of injury span beyond a single localized point, either due to the diffuse injury during the traumatic event or secondary damage because of the widely reaching inflammatory changes in the whole CNS^257,258^.

A specific type of astrocyte lineage cells differentiated from GRPs using BMP4, but not CNTF, stimulated functional motor recovery and had a protective effect on axotomised neurons^259–261^ in rats upon transplantation after SCI, without enhancing pain fiber sprouting (which presented a concern with some cell transplantation therapies in SCI^262^). Interestingly, the same type of astrocytes (GRP-derived astrocytes, GDA^BMP4^) was also shown to be protective in a rat model of PD^151^ (Table 2). On the other hand, GRPs differentiated in the presence of CNTF specifically promoted mechanical allodynia and thermal hyperalgesia, which correlated with the increased pain fiber outgrowth^260^, providing key evidence of astrocytic lineage heterogeneity and offering at least partial explanation for inconsistent success of astrocyte transplantation in other studies^262^ (Tables 5 & 6).

**Table 5.** Astrocyte Transplantation Strategies for the Treatment of SCI & TBI.

Disease or disease model and age at transplantation	Type of astrocyte-lineage cells or precursors transplanted	Site of transplantation within the CNS	Endpoint/treatment duration	Outcome summary	References
SCI—L5 dorsal root crush, albino rats, adult	Immature astrocytes from E16-18 fetal rat spinal cords, cultured for 2 days, plated on nitrocellulose Millipore inserts (‘pennants’) for further 1–2 days before insertion	Dorsal root entry zone at the CNS-PNS interface, implanted at the time of injury	Approx. 3 weeks	Enhanced but variable fiber regeneration into the spinal cord gray matter, attenuated inflammatory response, astrocytic coating of the implant prevented hemorrhage and cavitation.	Kliot et al.^263^
SCI—C3 fasciculus gracilis (carries sensory input from caudal parts of the body) aspiration, male Sprague-Dawley rats, adult	Cultured astrocytes from E14 fetal rat spinal cords, or whole pieces of E14 fetal rat spinal cord	C3 fasciculus gracilis area, implanted at the time of injury	Up to 90 days	Whole fetal spinal cord grafts improved hindlimb function; astrocytes from such grafts migrated into the nucleus gracilis of the medulla and prevented neuronal atrophy. Pre-cultured astrocytes also migrated to the medulla, but provided no neuroprotection, and exacerbated hindlimb dysfunction.	Bernstein and Goldberg^264^
SCI—hemisection of the dorsal L3 spinal cord, female Sprague-Dawley rats, adult	Cultured (>2 weeks) astrocytes from newborn rat cerebral cortex, either in HBSS solution or grown in gelfoam for 3 days	Dorsal L3 spinal cord, implanted at the time of injury	2 weeks to 2 months	Astrocytes in suspension and gelfoam reduced scarring and potentially increased neurofilament outgrowth; implanted astrocytes are capable of migrating.	Wang et al.^265^
SCI—focal infarcts at dorsal L13, male PVG rats, adult	(1) Neonatal PVG rat forebrain astrocytes cultured for 7 days (A1 astrocytes); (2) Neonatal kitten forebrain astrocytes cultured for 7 days; (3) CG-4 (bipotential rat glial progenitor cell line) -differentiated astrocytes (A2 astrocytes)	Dorsal L13 spinal cord, 3 days after the injury	4 weeks	Cultured rat astrocytes were the most prone to clumping and only filled 25% of the post-injury cyst; feline astrocytes and CG-4-differentiated astrocytes filled the cyst with fine processes more uniformly. CG-4-differentiated astrocytes increased vascularization, promoted some axonal sparing and potentially stimulated ECM production. No significant effect on axonal regeneration.	Olby and Blakemore^266^
SCI—bilateral dorsal hemisection of T8-9 spinal cord, female Wistar rats, young adult	Cultured cortical neonatal (P3) rat astrocytes (type 1) in collagen type I; cultured in collagen overnight prior to transplantation; 2mm wide section	Thoracic spinal cord, implanted at the time of injury	28 days / 4 weeks	Significant increase in neuronal process ingrowth into the astrocyte-containing implant from both rostral and caudal areas of the graft compared to collagen graft only; temporary motor improvements as assessed by the open-field locomotion analysis and crossing a walkway / catwalk analysis. Little astrocyte migration outside of the implant.	Joosten et al.^267^
SCI—complete section of T8 spinal cord, female Fischer 344 rats, adult	Cultured cortical astrocytes from adult female Fischer 344 rats from the same inbred as the host animals, GFP-labeled using a lentiviral vector	T11 spinal cord, 7 days after injury	6 weeks	Transplanted astrocytes migrated and accumulated at the site of injury; transgene was downregulated from approx. 80 to 7% over 6 weeks—to consider for gene therapy development.	Pencalet et al.^268^
SCI—dorsal column unilateral transection at C1-2, or rubrospinal tract unilateral transection at C3-4; female Sprague-Dawley or Fischer 344 rats, 3 months-old (adult)	(GRP, A2B5+)-GDAs: GRPs isolated from E13.5 Fischer 344 transgenic (expressing human placental alkaline phosphatase) rat embryos, differentiated by exposure to BMP4 (GDA^BMP^, type 1 astrocytes); GFP-labeled adult mouse DRG neuron implantation caudal to the injury site to assess axonal outgrowth in some animal groups	6 different areas of injection per lesion site, implanted at the time of injury	4 days to 5 weeks, depending on the test	Robust axon growth across lesion sites bridged with GDAs^BMP^, suppressed red nucleus neuron atrophy, reduced astrogliosis, increased linear alignment of host astrocytes at the lesion margin, delayed expression of inhibitory proteoglycans, recovery of locomotor functions as assessed by the grid-walk test—all seen after transplantation of GDAs^BMP^, but not GRPs. Greater variation in lesion size and margin morphology in Fischer 344 rats.	Davies et al.^259^
SCI—as above, female Sprague-Dawley rats, 3 months-old (adult)	GDAs differentiated from GRPs, either as above by treatment with BMP4 (GDA^BMP^), or by treatment with CNTF (GDA^CNTF^); GFP-labeled adult mouse DRG neuron implantation caudal to the injury site to assess axonal outgrowth in some animal groups	As above	As above	GDAs^BMP^, but not GDAs^CNTF^ or non-differentiated GRPs, support axonal regeneration and behavioral recovery as described above; GDAs^CNTF^ or non-differentiated GRPs, but not GDAs^BMP^, promote CGRP immunoreactive nociceptive c-fiber sprouting which results in mechanical allodynia and thermal hyperalgesia.	Davies et al.^260^
SCI—C3-4 unilateral transection, female Sprague-Dawley rats, 3 months-old (adult)	Human GDAs^BMP^ and GDAs^CNTF^, differentiated from human spinal cord glial precursors (9 week-gestational age)	As above	3 days to 5 weeks	GDAs^BMP^, but not GDAs^CNTF^, promoted axon ingrowth into the injury site and locomotor recovery, as assessed by the grid-walk test. These cells also expressed higher levels of GDNF, GLT1, and Connexin 43.	Davies et al.^261^
SCI—T9-10 moderate contusion injury, female Sprague-Dawley rats, 8 weeks-old (young adult)	GFP-labeled mouse iPSCs were differentiated into astrocytes through neurosphere and neural stem cell stages by treatment with FBS	T9-10, 3 or 7 days post-injury	Up to 8 weeks	Transplanted astrocytes extended processes longitudinally, but no motor improvement was observed; increased sensitivity to mechanical stimuli.	Hayashi et al.^269^
SCI—T10 contusion, female athymic rats, adult	Human brain-derived GRPs (18-to 24-week-gestational age); or human GDAs^BMP^ differentiated from GRPs	In and around (2mm rostral and caudal) the injury site, 9 days post-injury	Up to 8 weeks	Majority of transplanted human GRPs differentiated into astrocytes with some oligodendrocyte differentiation. Both GRP and GDA^BMP^ grafts reduced cyst and scar formation. GRPs attenuated hyperactive bladder reflexes; GDA^BMP^ graft improved sensory function of the hindpaw. No allodynia and pain and no locomotor improvement were observed, modest decrease in motor performance (grid-walk) upon GDAs^BMP^ transplantation.	Jin et al.^270^
SCI—C4-5 small excision of the right dorsal column, female Sprague-Dawley rats, adult	1) Rat GRPs were generated from spinal cord and cortices via 3 different methods; 2) Human GRPs were derived from the brain tissue (18- to 24-week-gestational age); 3) Astrocytes were differentiated from GRPs through treatment with either FBS, BMP4, or CNTF	C4-5, implanted at the time of injury	3 weeks	Differentiated astrocytes remained phenotypically plastic. Majority of engrafted cells differentiated into astrocytes. All grafts supported axonal growth; sensory axons could grow into but not out of the graft area.	Haas et al.^271^
SCI—T9 moderate to severe contusion, Fischer 344 rats, adult	GRPs from E14 Fischer 344 rat spinal cord were differentiated into GDAs by exposure to BMP4; GDAs were transduced with D15A multi-neurotrophin using a lentiviral vector	In and around the injury site, 8 or 9 days post-injury	Up to 8 weeks	Most grafted GRPs differentiated into astrocytes with some differentiating into oligodendroglial lineage cells. D15A-GDAs, but not GRPs or GDAs, increased spared white matter and promoted motor recovery assessed by the open-field locomotor test. No allodynia was detected.	Fan et al.^272^
SCI—C4-5 unilateral excision severing the right dorsal column, female athymic rats, adult	Human GRPs were derived from the brain tissue (20- to 21-week-gestational age); GDAs were differentiated through exposure to BMP4 or CNTF	C4-5, implanted at the time of injury	5 weeks	Each of the grafts (GDA^BMP^ or GDA^CNTF^) supported sensory axon outgrowth into, but not out of the graft. Pre-differentiated astrocytes retained morphologic and phenotypic plasticity.	Haas and Fischer^273^
SCI—C4-5 unilateral excision severing the right dorsal column, female Sprague-Dawley rats, adult	Human GRPs were derived from the brain tissue (20- to 21-week-gestational age) from frozen stocks	As above	As above	See above	Haas and Fischer^273^
SCI—T8 contusion, female Sprague-Dawley rats, adult	Rat GRPs were derived from E13.5 spinal cords, and GDAs were differentiated through exposure to BMP4; Injected alone or in combination with human recombinant decorin (hr decorin)	Around the injury site, implanted at the time of injury	4 weeks (28 days)	Both GDAs^BMP^ and GDAs^BMP^ + hr decorin reduced glial scar formation, filled the injury-induced cavity, enhanced linear alignment of astrocytic processes, increased motor and sensory axon regeneration, improved hindlimb motor function. GDA^BMP^ + hr decorin graft was the most efficient as inhibiting the inflammatory response.	Wu et al.^274^
SCI—C1-2 right dorsal column transection, female Sprague-Dawley rats, adult	Fischer 344 rat GRPs were derived from E13.5 spinal cords, and GDAs were differentiated through exposure to BMP4	Around the injury site, implanted at the time of injury	7 days	GDA^BMP^ graft promoted significant axonal growth into the lesion center. Knock-down of periostin (POSTN), a secreted protein, by shRNA diminished the axonal regeneration.	Shih et al.^275^
SCI—C2 hemisection, female Sprague-Dawley rats, adult	Fischer 344 rat GRPs were derived from E13.5 spinal cords	C2, implanted at the time of injury	5 weeks (35 days)	GRPs efficiently differentiated into astrocytes, reduced the macrophage response, stimulated regeneration of ventral respiratory group axons and sprouting of spared fibers, and protected diaphragm function. Notably, re-growing axons did not exit the graft site, suggesting that axonal recovery was probably not responsible for the functional effect.	Goulão et al.^8^
TBI—left frontal cortex defects were created with a scalpel, Sprague-Dawley rats, 12 weeks-old (adult)	hUC-MSCs co-grafted with astrocytes (region unspecified) from P1-2 Sprague-Dawley rats activated by 1ng/ml LPS 6h prior to use; transplanted in a peptide hydrogel R-B-SPH scaffold – a self-assembling peptide hydrogel enriched with a BDNF protein-derived sequence	Into the injury site, implanted at the time of injury	Up to 8 weeks	Presence of activated astrocytes increased BDNF secretion and hUC-MSC proliferation within the graft, promoted neuronal differentiation of hUC-MSC, and enhanced neuronal network reconstitution.	Shi et al.^276^
TBI—unilateral controlled cortical impact (CCI) that produced a moderate injury, male Sprague-Dawley rats, 3 months-old (adult)	GFP-expressing murine ESCs were differentiated into astrocytes in the presence of CNTF; GABAergic neuronal cells differentiated from the same ESCs and growth arrested stromal cells (bone marrow stromal fibroblasts) were used in parallel transplantations	2 sites in the injured cortex around the injury, 7 days following injury	Up to 6 weeks	ESC-derived GABAergic neurons, but not ESC-derived GDAs^CNTF^ nor stromal cells, improved sensorimotor recovery.	Becerra et al.^277^
TBI—lesion of the right motor cortex with probe pre-cooled in liquid nitrogen, male Sprague-Dawley rats, 7 weeks-old (adult)	Astrocyte-enriched mixed glial cultures were prepared from cerebral cortices of newborn rats and grown in FBS; cortical neurons from E16 rat embryos were used in parallel transplantations	Into the injured cortex, 5 days following injury	Up to 6 weeks	Glial graft significantly increased body weight and promoted motor recovery (assessed via the rotarod test) compared to the neuronal graft; both glial and neuronal grafts reduced the injury area.	Quan et al.^278^

SCI: spinal cord injury; TBI: traumatic brain injury; CNS: central nervous system; ECM: extracellular matrix; GRP: glial-restricted precursor; GDAs: Glial precursor derived astrocytes; CGRP: calcitonin-gene-related peptide; hUC-MSCs: Human umbilical cord mesenchymal stem cells; PNS: peripheral nervous system; BMP4: bone morphogenic protein 4; CNTF: ciliary neurotrophic factor; HBSS: Hanks’ balanced salt solution; FBS: fetal bovine serum; DRG: dorsal root ganglia; iPSCs: induced pluripotent stem cells; ESCs: embryonic stem cells; LPS: lipopolysaccharide; BDNF: brain-derived neurotrophic factor.

**Table 6.** Various Methods Employed to Generate BMP4-Differentiated Astrocytes for Transplantation.

Cell type of origin	BMP4 concentration	Duration of BMP4 treatment	Condition treated by transplantation and main outcome	References
Human induced PSCs (hiPSCs)	10 ng/ml (+ 10 ng/ml LIF)	20 days	ALS model—modest lifespan and motor improvement	Kondo et al.^73^
Rat (Fisher 344 hPAP transgenic) GRPs from the spinal cord of E13.5 embryos	10 ng/ml (+ 1 ng/ml bFGF)	7 days - ?	PD model—functional improvement, neuroprotection	Proschel et al.^151^
Human GRPs from the rostral neural tube of 9- to 10-week-old embryos	10 ng/ml	As above	As above	Proschel et al.^151^
NPCs from ubiquitous eGFP-expressing mouse cerebral cortex	10 ng/ml	4 days (96 h)	FTD model—reduced neuronal loss similarly to NPCs	Hampton et al.^206^
Rat (Fisher 344 hPAP transgenic) GRPs from the spinal cord of E13.5 embryos, A2B5-positive	10 ng/ml	7 days	SCI—functional improvement	Davies et al.^259^
As above	10 ng/ml	7 days	SCI—functional improvement which was not seen with GRP-differentiated astrocytes (GDAs) differentiated in the presence of CNTF (GDA^CNTF^)	Davies et al.^260^
Human glial progenitor cells (hGPCs) from spinal cords of 9 weeks-old embryos, A2B5-positive	20 ng/ml	7 days	SCI—functional improvement which was not seen with astrocytes differentiated in the presence of CNTF	Davies et al.^261^
Human GRPs from the brain tissue of 18- to 24-week-old embryos	50 ng/ml	5 days	SCI—improved sensory function, modestly decreased motor performance	Jin et al.^270^
Rat GRPs from the spinal cord generated via 3 different methods; Or human GRPs from the brain tissue of 18- to 24-week-old embryos	10 ng/ml, or 50 ng/ml in select experiments	6 days	SCI—supported axonal growth, remained phenotypically plastic despite pre-differentiation	Haas et al.^271^
Rat GRPs (Fischer 344) from E14 spinal cord, A2B5-positive	20 ng/ml	3 days	SCI—only GDAs that were transduced to express D15A neurotrophin showed positive effects on motor recovery and white matter sparing	Fan et al.^272^
Human GRPs from the brain tissue of 20- to 21-week-old embryos	10 ng/ml	10 days	SCI—supported sensory axon outgrowth, remained phenotypically plastic despite pre-differentiation	Haas and Fischer^273^
Rat GRPs (Sprague-Dawley) from E13.5 spinal cord	10 ng/ml	7 days	SCI—improved motor function	Wu et al.^274^
Rat GRPs (Fisher 344 hPAP transgenic) from E13.5 spinal cord	10 ng/ml (+1 ng/ml bFGF)	6 days	SCI—axonal regeneration dependent on periostin secretion	Shih et al.^275^

BMP4: bone morphogenic protein 4; ALS: amyotrophic lateral sclerosis; GRPs: glial-restricted precursors; PD: Parkinson’s disease; NPCs: Neural precursor cells; FTD: frontotemporal dementia; SCI: spinal cord injury; GDAs: Glial precursor derived astrocytes; LIF: leukemia inhibitory factor; hPAP: human placental alkaline phosphatase; bFGF: basic fibroblast growth factor; eGFP: enhanced green fluorescent protein; CNTF: ciliary neurotrophic factor.

One obvious way in which an astrocyte graft can alleviate the symptoms associated with trauma is by filling up the cavity and replenishing lost cells in the damaged area. Lesioned axons fail to send new projections through a fluid-filled cyst that can result post-trauma, while astrocytes, which can be engrafted in a gel biomaterial such as collagen^267^, contribute to restoring a more physiological ECM composition. Even though some earlier reports considered astrocytes to be the primary source of inhibitory ECM components that are detrimental to axonal regrowth, new evidence strongly suggests that astrocytic presence at the lesion site is crucial for healing^279,280^. Furthermore, healthy transplanted astrocytes were able to delay expression of inhibitory ECM molecules when engrafted during early post-lesion stages, suppress astrogliosis of the resident cell populations, and re-organize the injured tissue to potentially make the scar border more permissive to axonal growth^259,274^.

In addition to the primary damage to the tissue at the time of trauma^281^, secondary axonal injury leading to neuronal body atrophy and apoptosis^282–285^, also occurs. Astrocyte-mediated protection of myelin sheaths surrounding damaged areas can be prompted by a combination of connexin cell-to-cell contacts^22^ and secreted factors such as thrombin protease inhibitors^23^, ATP, and LIF^286^. Additionally, astrocyte-derived CXCL10 can promote microglia-mediated phagocytosis of myelin debris^32^, which is essential for remyelination^287^. Astrocytes can also support surviving neurons and encourage new neurite outgrowth by providing neurotrophic cues, both secreted (such as BDNF and NT-3^262,272^) and cell surface bound (such as Eph receptors^288^).

Purinergic^289^ and glutamatergic^290^ excitotoxicity due to excessive release of ATP or glutamate, respectively, represent another feature of traumatic CNS injury, and therefore blockade of these pathways ameliorates secondary cell death and function loss after trauma. Oxidative stress also contributes to the cell death following injury as rapid generation of reactive oxygen and nitrogen species exacerbate excitotoxicity and damage mitochondria^291^. The ability of astrocytic syncytium to buffer excessive ATP and glutamate as well as to supply glutathione and other antioxidants were reviewed above in the context of other conditions, and it is likely that similar canonical pathways could be tapped into in the context of SCI and TBI.

Another important aspect of the functional recovery from trauma is restoration of the BBB and vascular supply to the injured area, in which astrocytes play a crucial role. Indeed, transplanted human iPSC-derived astrocytes were found to interact closely with the blood vessels^292^, and were able to increase vascularization of the lesioned area^266,263^.

Inconsistency of the astrocyte transplantation success between different models of SCI is an important concern, and several reasons for this phenomenon were suggested^262^. First, the type of injury, location, and severity are likely to be important determinants of what extent of functional recovery can be reasonably expected. For example, some studies on SCI rodent models have demonstrated locomotor recovery^259^ while others presented sensory recovery with no motor improvement^270^. It is possible that the cell numbers, density, and vehicle used for astrocyte delivery (e.g. collagen-based gel or liquid medium) can play a role in determining the outcome (Table 5).

Second, the time of transplantation post-injury is a key variable that needs to be assessed. It has been suggested that inflammation and reactive astrogliosis are beneficial at the early healing stages^293,294^ (e.g. due to recruiting microglia to promote debris clearance) and therefore not allowing the natural protective response of resident astrocytes to develop could be detrimental. In fact, delayed transplantation studies are more likely to be relevant to the real clinical cases where many patients would seek treatment days, weeks, or years after the initial injury.

Finally, it is obvious from the published SCI studies that the type of astrocytes to be transplanted (e.g. mature vs embryonic, A1 vs A2 type, BMP4- or CNTF-differentiated GDAs, plus regional differences) plays a crucial role in determining the likelihood of recovery. For instance, different subtypes of astrocytes exhibit differential tropism for encouraging support of specific neuronal fibers as demonstrated by the GDAs^BMP4^, which specifically promoted motor axon outgrowth, while GDA^CNTF^ mostly enhanced extension of nociceptive calcitonin-gene-related peptide (CGRP) c-fibers^260^. Several different methods used to differentiate rodent and human astrocytes using BMP4 are summarized in Table 6. The ability of transplanted GDAs to maintain their beneficial phenotype long-term in a hostile niche has been debated; however, since their effect on functional recovery may depend more on the initial positive effect on the host tissue than the continued presence of these cells^259^, cell pre-differentiation could still be a viable therapeutic approach. In an attempt to ensure that GDA^BMP4^ retain their beneficial phenotype and continue providing the trophic factors even under unfavorable conditions, bioengineering approaches have been employed: retroviral transduction of these cells with D15A (an engineered neurotrophin that combines NT-3 and BDNF activities) enhanced the ability of the graft to improve locomotion in rats with SCI^272^. Effective healthy astrocyte replacement in traumatic CNS injury would help a patient to regain the lost neurological function fully or partially, and/or reduce the burden of neuropathic pain, thereby increasing their capacity for independent living.

### Ischemia/Stroke

Cerebral ischemia, most commonly resulting from stroke, is one of the leading causes of disability and death worldwide, which is associated with devastating losses of various neurological functions and increased risk of dementia for the patients^295^. The incidence of stroke ranges from 95 to 290 new cases per 100 000 people per year, and 13% to 35% of cases lead to death within 1 month^296^. Stroke is more common in older patients with the mean age of occurrence around 70 years^297^, but up to 15% of stroke patients are young adults, for whom personal and economic implications of the life-long disability are much greater^298^. While some genetic factors such as history of familial hypertension predispose certain people to stroke, a combination of environmental factors and lifestyle has a large impact on the probability of development of this pathology, which include obesity, lack of exercise, psychological stress, and smoking^299^.

Ischaemic stroke is commonly caused by an acute blockade of a cerebral artery by a thrombus that results in the cessation of blood supply to particular brain areas, leading to cell death at the center of the infarction within minutes of the onset. As in the case of traumatic injury, specific location of the ischaemic event determines the extent and nature of the disability that is likely to result from it.

To rescue memory deficits in the aftermath of an ischaemic stroke in rats, co-transplantation of NSCs alongside astrocytes, brain microvascular endothelial cells (BMECs), or both, has been performed. While NSC graft alone did not affect rats’ cognitive performance, double (astrocytes+NSCs or BMECs+NSCs) or triple transplantation led to significant improvements^300^. Another approach to employ astrocytic transplantation to improve cognitive and motor function after ischemia in rats involved knocking down astrocytic CDK5 prior to engraftment^301,302^ (Table 7).

**Table 7.** Astrocyte Transplantation Strategies for the Treatment of Ischaemic Stroke.

Disease or disease model and age at transplantation	Type of astrocyte-lineage cells or precursors transplanted	Site of transplantation within the CNS	Endpoint/treatment duration	Outcome summary	References
Ischemia—middle cerebral artery occlusion and reperfusion, male Sprague-Dawley rats, adult	Astrocytes were derived from neonatal Sprague-Dawley rat cortices and cultured in FBS-containing medium; NSCs and brain microvascular endothelial cells (BMECs) were also isolated from newborn rat cortices and used in parallel transplantations	Hippocampal CA1, 12 days after injury	8 weeks	Co-transplantation of all 3 cell types was the most efficient at memory improvement as assessed by the Morris water maze test. NSCs alone had no effect on memory.	Cai et al.^300^
Global cerebral ischemia—permanent left carotid artery occlusion, temporary right carotid artery occlusion (20min) and reperfusion, male Wistar albino rats, 3 months-old (adult)	Astrocytes were obtained from the P1-2 rat cerebral cortices; CDK5 microRNA knock-down (CDK5-KD) was employed	Somatosensory cortex, immediately after the injury	24 h to 15 days	CDK5-KD astrocytes were significantly more efficient at rescuing cognitive and motor impairment after ischemia as assessed by rotarod and neurological evaluation, prevented loss of neurons, increased an endothelial cell marker expression, and upregulated BDNF levels.	Becerra-Calixto and Cardona-Gómez^301^
Global cerebral ischemia—as above	As above	As above	4 months	CDK5-KD astrocytes prevented loss of host astrocyte and neurons post-injury, protected endothelial cells, enhanced BBB recovery, enhanced BDNF production by endogenous astrocytes, and improved neurological and motor (rotarod) performance.	Becerra-Calixto et al.^302^

CNS: central nervous system; NSCs: neural stem cells; BBB: blood-brain barrier; FBS: fetal bovine serum; BDNF: brain-derived neurotrophic factor; CDK5: cyclin-dependent kinase 5.

CDK5 hyperactivation has been observed in several disorders including ischemia, AD, and ALS, which results from the calcium-dependent p35 to p25 cleavage^303,304^. It was therefore hypothesized that preventing CDK5 activation can render astrocytes in their non-pathologically activated state. Indeed, CDK5 knock-down increased the ability of transplanted astrocytes to prevent local cell loss, protect the BBB, and ultimately improve neurological scores as well as locomotor recovery^301,302^.

Consistent with the detrimental role of calcium-mediated hyperactivation of CDK5 in ischaemic stroke, abnormally increased levels of astrocytic calcium were seen in reactive astrocytes following the insult, which result from the extracellular calcium entry^305^ or release of calcium from the intracellular stores^306^. Interestingly, there appears to be a level of heterogeneity in the roles played by astrocytic calcium responses, which may stem from the spatial (endfeet vs soma), or temporal (e.g. frequency of calcium oscillations^307^), location of signaling after the ischaemic insult. For instance, release of calcium in the astrocytic endfeet, but not cell bodies, enhances vasodilation to improve brain microcirculation, which may be protective in ischemia^308^. At the same time, mGluR activation that promotes rhythmic calcium oscillations leads to the pathological swelling in astrocytes^309^, but also has cytoprotective effects on white matter astrocytes^310^. Decreasing CDK5 may specifically dampen the detrimental effects of calcium dyshomeostasis under ischaemic conditions while allowing the protective signals to remain.

Excitotoxicity is a common feature of many pathologies, and ischemia is no exception. Excessive calcium oscillations triggered in astrocytes by ischaemic insults were associated with CaMKII inhibition, which led to the reduced ability of astrocytes to buffer extracellular glutamate as well as an increase in ATP release^311^. This therefore perpetuates the excitotoxic cycle by acting on astrocytic glutamatergic receptors—the expression of which can be stimulated by hypoxic conditions^312^, and purinergic receptors, which further dysregulates intracellular calcium levels. Damage to astrocytic mitochondria was observed in other studies as well^313,314^, which further contributes to the BBB breakdown^315^ and GLT1 glutamate transporter downregulation^316^. Certain regional populations of astrocytes, such as those found in the hippocampus, were found to be specifically vulnerable to ischemia-induced mitochondrial damage and oxidative stress, leading to the early loss of glutamate transporters^317^ which further impacts neighboring hippocampal neurons. This may be the underlying reason of frequently observed memory deficits following stroke.

Inflammatory molecules such as interleukin-1β (IL-1β) are known to contribute to cell damage in stroke, and one of the inflammation-induced mechanisms of damage involves activation of p38/stress-activated protein kinase 2 (p38/SAPK2), leading to gap junctional closure. Accordingly, inhibition of p38/SAPK2 pathway was found to reduce the area of ischaemic lesion^318^, suggesting that the gap junctional communication is crucial in preventing the injury spread. Moreover, human marrow stromal cell (hMSC) transplantation aided neurological recovery in rats following stroke, and these cells were shown to be able to release soluble factors that upregulate gap junctional communication in astrocytes through activation of PI3K/Akt pathway^319^. At the same time, blockade of hemichannels composed of connexin 43, the opening of which is potentiated under inflammatory and hypoxic conditions, had beneficial effects through downregulation of inflammatory cytokine production and microglial activation alongside prevention of release of ROS, ATP, and glutamate^320–322^.

Even though neurons were thought to be more vulnerable to ischemia than astrocytes, it is becoming clearer that both cell types suffer from pathological changes that commonly result in cell death^323^. Transplanted astrocytes can therefore replenish the resident panglial network, support the BBB, and improve the blood flow to the brain, and supply neuro- and astro-protective factors such as erythropoietin^309,324^. Effective healthy astrocyte replacement in the ischaemic stroke could, similarly to the traumatic injury to the CNS, help restore the compromised functionality, the exact nature of which would depend on the affected region of the brain.

## Overview

Astrocyte biology is an expanding topic of brain science research with the functions of these cells continually expanding from neuronal supporters to active partners at synapses to cellular hubs integrating signals and influencing functions of all major brain cell types. This review explores some prominent examples of astrocyte transplantation strategies with promising functional benefits in translational models of ALS, PD, AD, HD, ischemia, and traumatic CNS injury, and a pioneering clinical trial with human ALS patients points at a clear therapeutic interest of these studies.

Considering that many more brain and spinal cord disorders are found to be associated with astrocytic abnormalities, it is likely that the insights gained from these experimental transplantation strategies can be expanded to treatments of other diseases. For example, astrocytic abnormalities were found to precede demyelination in multiple sclerosis^325^, and astrocytes carrying mutations associated with a higher risk of autism were found to inflict damage on healthy neurons co-cultured with these mutant cells^30^. Therefore, replenishing healthy astrocyte pools can alleviate symptoms of more than one condition, especially considering that, despite different aetiologies, many disorders share key pathological alterations in astrocyte biology, demonstrated in this review. Transplantation of healthy cells and/or tapping into the canonical pathway onto which multiple pathologies converge (e.g. inflammation-induced astrocytosis, mitochondrial stress, or astrocytic syncytium-mediated calcium, ATP, and glutamate buffering) represents a robust and versatile approach.

Autologous cell sources are especially promising in human astrocyte transplantation therapies due to the lack of ethical concerns and minimized risk of rejection. Enteric glia have proven beneficial in an AD model upon engraftment in the brain^205^; however, recent studies point toward early dysfunction of these cells in degenerative disorders such as AD and PD^326^. Moreover, in case a patient harbors a mutation in all somatic cells that predisposes one to a certain disorder, even reprogramming of patient own fibroblasts into seemingly healthy astrocyte-lineage cells may result in inherently diseased astrocytes devoid of beneficial properties^81^. Gene-editing technologies such as CRISPR could be employed to correct known mutations associated with familial forms of diseases prior to implantation of the cell graft. Increased expression of transcription factors and neurotrophins, or knock-down of factors known to be associated with pathologically reactive astrocyte states, which is likely to shift the astrocytic phenotype toward a less inflammatory state, could also be additionally employed^152,272,301,302^.

One interesting point to consider during cellular reprogramming is regional heterogeneity of vulnerability to particular diseases—it is becoming increasingly apparent that brain cells in different brain regions exhibit different levels of susceptibility to pathologies associated with PD, AD, ALS, or ischaemic challenge. At the same time, progress has been made in the field of regional patterning of reprogrammed astrocytes^327–329^. Thence, should the transplantation therapy aim to replace the cells that correspond to the most vulnerable and affected region since these cells are more likely to be lost or severely affected by the disease, or should it supply those cells exhibiting a more “robust” regional phenotype?

Astrocytes, once matured and integrated in the brain, are not migratory^330,331^, and they retain many aspects of their regional identity *in vitro*^332^; hence, they can be expected to retain important aspects of such identity after transplantation as well. One study has shown that grafting midbrain, and not cortical, astrocytes were more beneficial in a PD model, arguing that the midbrain cells are more naturally adapted to supporting dopaminergic neurons^152^. One limitation of this study is that these astrocytes were transplanted into the rat striata, while no astrocytes from striatal origin were engrafted, and no transplantation into the midbrain was explored. Another good example of this point can be found in the field of ALS research. While cervical spinal cord-localized transplantation of rodent spinal cord-derived astrocyte precursors was beneficial in a rat model of ALS^71^, human forebrain-derived astrocyte precursors showed no protective effects in a mouse ALS model^72^. Even though both studies were conducted by the same group and used the same overall approach, the number of variables makes it difficult to compare these studies directly (e.g. mouse vs rat model, rat vs human cells); nevertheless, it is possible that the spinal cord astrocytes are specifically more apt to integrate into the spinal cord astrocytic and neuronal networks and restore relevant functions. Another corroboration of this idea comes from a study of the corticospinal tract (CST) axonal growth in neonatal rat spinal cords—when nitrocellulose inserts were placed in the cords to deflect the normal CST growth path, only spinal cord astrocytes grown on the inserts were able to support CST axon growth as opposed to cortical astrocytes^333^. On the other hand, enteric astrocytes have proven protective in an AD model when transplanted into the brain^205^, even though no comparison with other regional types was offered by that study. In addition, it has been suggested that astrocytes from the brain regions representing axonal targets, such as cortical astrocytes for hippocampal neurones, could promote axonal outgrowth more efficiently that astrocytes from the same brain regions as neurones, or unrelated regions of the brain.^334^ This property could be useful in cases where prompt neurite extension is desirable, such as after acute SCI, but the long-term effects of non-native astrocyte subtypes in a particular region of the brain are not known. More research is needed to address this issue in greater depth.

Engraftment of neurons, as described above^2–5^, and other cell types such as brain microvascular endothelial cells (BMECs)^300^ and oligodendrocytes^335^ also provide valuable therapeutic strategies, and, in fact, olidogendroglial differentiation of GRPs (in addition to astrocytic differentiation) can be at least partially responsible for the success of the GRP engraftment in some of the studies discussed above^8,270^.

Astrocytic transplantation can be conducted in parallel with engraftment of other cell lineages, as explored in several studies^152,300^, in order to maximize the beneficial effects, since even in those CNS disorders where pathology is commonly found most prominently in one cell type, multiple cellular perturbations are expected, and the cellular culprit of the pathology is not always obvious. Examples of that include the non-cell autonomous neuronal death in rodent models of ALS bearing mutant astrocytes^80–82^ and myelination pathology in Alexander’s disease caused by mutations in GFAP^336^—an intermediate filament protein associated with the astrocytic lineage.

But does cell transplantation merely alleviate disease symptoms? A fascinating study demonstrated that human glial precursors, which abundantly differentiate into astrocytes, when grafted into the mouse forebrain could integrate into the local cellular circuits and maintain their highly branched morphology, and also increase the number of synaptic contacts. Most importantly, these human cells enhanced the memory retention ability of these animals^337^ (Table 8).

**Table 8.** Astrocyte Transplantation in the Healthy Rodent Brain.

Disease or disease model and age at transplantation	Type of astrocyte-lineage cells or precursors transplanted	Site of transplantation within the central nervous system	Endpoint/treatment duration	Outcome summary	Reference
Healthy neonatal immunodeficient (rag1-/- or rag2-/-) mice	Human fetal glial progenitors extracted from the forebrain ventricular and subventricular zones (17- to 22-week-gestational age); murine glial progenitor cells were prepared from newborn pups in a similar fashion and used in parallel transplantations	Two locations within the forebrain, within a day of birth	2 weeks to 20 months	Glial precursors differentiated largely into astrocytes and retained hominid features (larger size, threefold faster calcium wave propagation); chimaeric mice containing human cells exhibited enhanced long-term potentiation (LTP) and learning, as assessed by Barnes maze test, object-location memory, and fear conditioning. No enhancement of memory was observed in animals who received murine grafts.	Han et al.^337^

Considering the accumulating data showing that attention-modifying and psycho-modulatory (e.g. antidepressants) drugs commonly used in humans, which used to be considered acting purely on neuronal networks, are increasingly recognized to also affect astrocyte biology^338^, and that glial precursors (abundantly differentiated into astrocytes) derived from schizophrenic patients were able to recapitulate certain key behavioral aspects of this pathology in rodents after transplantation^339^, the idea of treating more subtle psychological disorders and even enhancing cognitive abilities by modifying the phenotype and number of astrocytes in the human brain may become a realistic perspective. Perhaps attenuation of aging-related decline could represent a more immediately achievable goal as benefits of rejuvenation of the astrocytic niche have started being explored in aged animals^340^.

## Summary and Conclusions

Grafting of healthy astrocytes can slow down the disease progression and/or improve functional outcome in translational models of ALS, PD, AD, HD, ischemia, and traumatic CNS injury, and a human phase IIa clinical trial has shown a significant patient benefit in the case of ALS.

**Figure 1.**
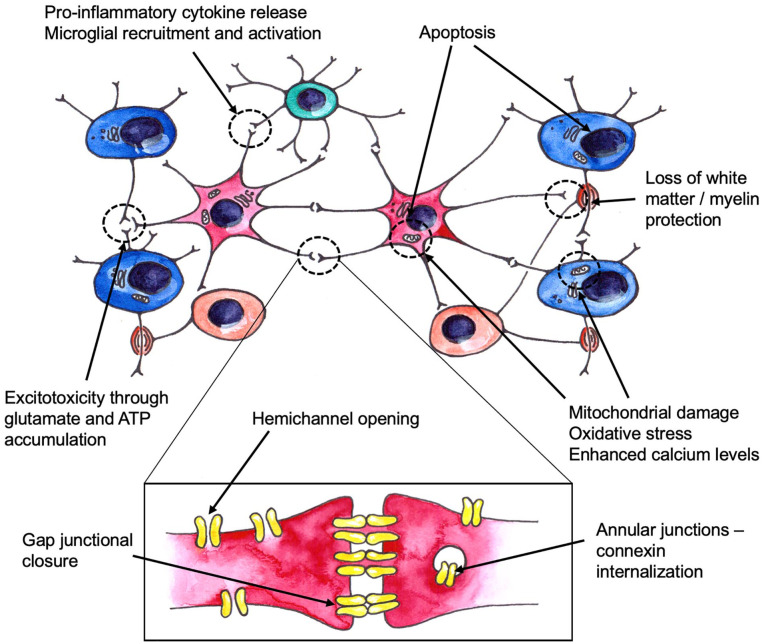
Brain cell network alterations in disease. Magenta—astrocytes, blue—neurons, orange—oligodendrocytes, green—microglia. ATP: adenosine triphosphate.

There are some common pathological features that are typically found in the diseased native astrocytes in these conditions that can lead to astrocyte atrophy and death, damage to surrounding neurons, loss of myelin, and recruitment of neurotoxic microglia (Fig. 1). These include:

1. Pro-inflammatory cytokine release profile (e.g. IL-6, TNFα, IL-1β) and loss of ability to release protective factors (e.g. BDNS, NT-3, GDNF);
2. Mitochondrial damage and increased ROS production, decreased ATP production;
3. Calcium signaling disturbance;
4. Connexin dysregulation including abnormal hemichannel opening;
5. Loss of glutamate transporters leading to excitotoxicity;
6. Loss of potassium buffering ability further leading to increased excitability of surrounding neurons;
7. Autophagy and proteasome deficits;
8. Apoptosis.

Transplanted astrocytes may therefore be able to (among other, disease-specific benefits):

1. Replace the apoptotic/necrotic astrocytes;
2. Reduce astrocytosis and inflammation;
3. Supply protective soluble (e.g. anti-inflammatory cytokines) and membrane-bound (e.g. Eph receptors, connexins) factors;
4. Reduce accumulation of toxic compounds including misfolded proteins and excessive glutamate, potassium, and calcium through lysosomal and proteasomal degradation and “glymphatic unit” clearance;
5. Act as stem cell-like cells with neurogenic potential.

Considering the emerging role of astrocytes in many other disorders, astrocyte transplantation is likely to become a more widespread therapeutic approach, alone or in combination with transplantation of other cell types such as neurons or NSCs.

The source of the cells suitable for transplantation needs to be determined with caution. Scalability and reproducibility issues with the available cell material may be a limitation of this therapeutic approach when scaling up from the experimental settings into the clinical trials and wider administration to the patients. There are three main types of cell sources that could be used for this purpose that we have identified:

1. Astrocyte precursor from ethically derived from the human embryos. This source has been used e.g. for the derivation of stem cells in transplantation trials involving people with PD^3,1^;
2. Commercially available pre-differentiated cells such as AstroRx produced by Kadimastem for the treatment of ALS;
3. Autologous cell transplantation where fibroblasts or mesenchymal stem cells are harvested from each patient and differentiated into the astrocyte-lineage cells ready for transplantation.

While there is a certain advantage to using the latter type of cells that are patient-specific, and thereby minimize the potential rejection and the extent of immunosuppression needed, they present two considerable limitations. First, differentiation of fibroblasts or mesenchymal stem cells into astrocyte-lineage cells, especially with the addition of time to characterize the resulting cells, can take several months, therefore delaying the benefit to the patient. Second, the presence of the known and yet-unknown mutations and epigenetic modifications that contribute to the disease phenotype may persist in the reprogrammed cells. Related to this issue, the batch-to-batch variability would be high between the cell transplants influenced by the patient-specific characteristics, and faithful comparison of the treatment outcomes would be difficult.

The precursor cells derived from human embryos could be considered more reliable in terms of their true astrocytic phenotype when compared with the differentiated cells, the identity of which is typically determined by the expression of rather generic astrocyte markers including GFAP. At the same time, use of human embryonic material presents an obvious limitation in the quantity of cells that can be obtained and the ethical considerations of scaling the cell supply up. In addition, batch-to-batch variability related to the differences between the embryos remains.

With this in mind, commercially available pre-differentiated and pre-characterized cells such as AstroRx could prove the most practical for the clinical use, as corroborated by their success in the first astrocyte transplantation clinical trial. These cells can be expanded, pre-characterized, and cryopreserved—these processes are currently being standardized to make the cells more suitable for the clinic. Refining the astrocyte-generation technology by creating more brain-region specific astrocyte types can expand this approach to a more diverse range of brain conditions.

The following additional consideration could be taken into account before this treatment finds its way into clinic:

1. Any cell graft has a small risk of over-proliferation. Pre-differentiation of stem cells into committed astrocyte precursors can help reduce the risk of graft overgrowth;
2. Hostile microenvironment of the diseased/lesioned tissue can predispose initially healthy cells to adopt a reactive and detrimental phenotype. Engineering astrocytes to artificially express trophic factors such as D15A (a human-designed neurotrophin that combines NT-3 and BDNF activities^272^), transcription factors (Nurr1 and Foxa2^152^) that are known to promote a non-reactive phenotype, or suppressing factors such as CDK5 that enhance pathological reactivity^301,302^ can ensure long-term maintenance of a healthy phenotype even in the presence of unfavorable conditions. Additionally, other small molecule or gene therapy-based approaches can be employed alongside transplantation to reduce inflammatory responses. For instance, minocycline and COX-2 inhibitors suppress neuroinflammation and extend lifespan of ALS mice^341,342^; more specific astrocyte- and brain-targeted interventions can be developed in the near future.
